# Nutrition and Physical Exercise in Women

**DOI:** 10.3390/nu14142981

**Published:** 2022-07-21

**Authors:** Sílvia Rocha-Rodrigues, José Afonso, Mónica Sousa

**Affiliations:** 1 Escola Superior de Desporto e Lazer, Instituto Politécnico de Viana do Castelo, Rua Escola Industrial e Comercial de Nun’Alvares, 4900-347 Viana do Castelo, Portugal; 2 Tumour & Microenvironment Interactions Group, INEB—Institute of Biomedical Engineering, i3S-Instituto de Investigação e Inovação em Saúde, Universidade do Porto, Rua Alfredo Allen, 208, 4200-153 Porto, Portugal; 3 Centre for Research, Education, Innovation and Intervention in Sport (CIFI2D), Faculty of Sport, University of Porto, Rua Dr. Plácido Costa, 91, 4200-450 Porto, Portugal; jneves@fade.up.pt; 4 Nutrition and Lifestyle, NOVA Medical School, Faculdade de Ciências Médicas, NMS, FCM, Universidade Nova de Lisboa, Campo Mártires da Pátria, 130, 1169-056 Lisboa, Portugal; monica.sousa@nms.unl.pt; 5 CINTESIS-Center for Health Technology Services Research, NOVA Medical School, Faculdade de Ciências Médicas, NMS, FCM, Universidade NOVA de Lisboa, 1169-056 Lisboa, Portugal

While the benefits of nutrition and physical exercise are commonly studied separately, their concomitant integration has the potential to produce greater benefits in women than strategies focusing only on one or the other [1]. Studying the specificities of women in response to interventions is of the utmost importance for providing optimal healthcare and aids specifically designed guidelines that are better suited for women [1]. Women have a number of specificities that differentiate them from men, particularly the variations of sex steroid hormone, especially oestrogens and progestogens, which significantly impact women’s physiology [2,3]. Cumulative evidence demonstrates that the combination of healthy nutrient intake and regular physical exercise is a powerful lifestyle strategy that modulates lifelong health through its ability to improve body composition, sex-steroid hormones, and physical performance and prevent chronic diseases across the lifespan [1].

With this Special Issue on “Nutrient Intake and Physical Exercise as Modulators of Healthy Women”, we are honoured to contribute with important pieces of evidence for an integrational approach—nutrition and physical exercise—as a potential modulator of lifelong. It includes ten studies: eight articles and two narrative reviews. Please let us introduce the articles with a short summary.

The differences in substrate oxidation between men and women have been a topic of interest in the past few years. In this line, Nosaka et al. [4] performed a randomised, double-blind, placebo-controlled, three-arm, within-participants crossover trial during three 14-day interventions separated by two 14-day washout periods to analyse the impact of medium chain fatty acids supplementation on men and women aged 40–59 years. Women showed an increase in carbohydrate oxidation and oxygen uptake during the exercise trial after the C10R diet, while no changes in fatty acid oxidation were detected compared to men. Of note, the differences between sex on the type of substrate oxidation and on strategies to enhance oxidative pathways, both during exercise, are definitively topics that need further enlightenment.

In elderly women, Amirato et al. [5] found that 30 days of L-glutamine supplementation (10 g/day) and endurance and resistance exercise significantly improved glycemia control, plasma antioxidant capacity, and strength and power of knee muscles. Dechichi et al. [6] focused their studies on a very special phase in a woman’s life—menopause. The topic of women’s health during menopause has been brought to attention in the past few years, with isoflavones being tested as a substitute for endogenous oestrogens. In non-obese postmenopausal women, Dechichi et al. [6] aimed to study if the isoflavones supplementation had additive effects on aerobic and resistance exercise on resting and ambulatory blood pressure monitoring, and on blood pressure variability, in non-obese postmenopausal women. The authors reported no additional effects of isoflavone supplementation on these blood pressure-related variables.

In apparently healthy women, Waliko et al. [7] assessed whether rumination (a form of repetitive negative thinking) could lead to changes in eating behaviours (usually in a maladaptive direction). Overall, 188 women were recruited for the study (83% response rate), and rumination was partially associated with uncontrolled eating and emotional eating. The authors propose that targeting rumination could help in reducing uncontrolled and/or emotional eating. However, due to the nature of the study design, no causal inferences should be made. Moreover, the terminology of “predictors” that was used across the manuscript should be best interpreted as “correlates”. While it is possible that rumination increases the likelihood of maladaptive eating behaviours, it is also possible that maladaptive eating behaviours stimulate rumination. Still, breaking this potential positive feedback loop could help the subjects return to their healthier eating patterns; additionally, acting on rumination may provide an interesting strategy for achieving this intent.

In overweight or obese women with hypertension, Dos Santos Fechine et al. [8] found that 8-weeks of fibre supplementation (guar gum, NutraFlora, psyllium and microcrystalline cellulose) resulted in differential metabolite response when compared to a control group. Specifically, the experimental group observed an increase in peak intensity values of all three analysed metabolites, accompanied by a decrease in blood pressure. Overall, the results suggest that overweight and obese women with hypertension could benefit from the intake of mixed dietary fibre.

The term normal weight obesity (NWO) was firstly described by Lorenzo et al. [9] and was defined as a body mass index ≤ 25 kg/m^2^ but with an increased body fat percentage. The cut-offs used for body fat percentage have varied depending on the study population, sex, and ethnicity. NWO is a widespread public health issue that may be prevalent in up to one-third of individuals [10]. In NWO women, Haghighat et al. [11] reported that six-month of soy-enriched high-protein snack meals containing 50 g of soybean (vs. low-protein snacks containing 3.5 servings of fruits) improved body composition parameters, including increased skeletal muscle mass and reducing body mass, body fat percentage, and appetite levels. These findings underline the clinical applicability that consuming a soy-enriched high protein snack replacement may provide a practical approach to controlling energetic intake and improving body composition in cohorts with NWO.

Attention to preferred physical activities encourages participation in physical activity. Participation in physical activity is associated with a better body size perception but not in a consistent way. In this line, Hubert et al. [12] reported that collegiate women (*n* = 251; 74% white), who perceived greater body size, reported less liking of physical activity as well as less healthy dietary behaviours. Additionally, women whom both liked and engaged in physical activities had a lower body size perception and healthier diet quality.

In a prospective randomised trial, Bak-Sosnowaska et al. [13] found that all women aged 18 to 65 years with BMI ≥ 30 kg/m^2^) presented significant changes in the anthropometric parameters, namely decreased body weight, waist and hips circumference, as well as body mass index (BMI) and waist-to-hip ratio (WHR) after three months of endurance or endurance strength training intervention. Additionally, those alterations were associated with a better perception of the current figure and a lower level of concern about body shape, with more significance for the endurance training group compared to the endurance strength training group.

In Rocha-Rodrigues et al. [14]’s review, an overview of the mechanisms behind the relationship between menstrual cycle, exercise, and nutritional intake in women was made by exploring the roles of oestrogens and progestogens in response to exercise and how exercise impacts their regulation. Although some internal physiological parameters vary across the menstrual cycle, their impact on performance seems to be highly dependent from woman to woman, and the magnitude of effects tends to be residual or trivial at best. Moreover, the energy demands and nutritional intake in women in relation to hormonal fluctuations face similar difficulties, as higher energy expenditure in some phases of the menstrual cycle tends to be naturally compensated by an increase in dietary intake. Indeed, this is a promising field of research, but one where the search for populational trends may have to be replaced by highly individualised approaches due to the considerable heterogeneity and variability of responses.

The roles of diet and nutrition on gynaecological disorders were reviewed by Afrin et al. [15]. The evidence suggests that nutritional habits play a role in the development of gynaecological disorders, although it is not clear whether this relationship is associative or causative; if causative, it is unclear whether nutritional habits may contribute to causing such disorders or merely aggravate already existing disorders, or even if gynaecological disorders cause changes in nutritional habits. When gynaecological disorders are installed, varied diets (especially rich in fruits and vegetables), green tea, vitamin D, and other resources may help achieve better development of the disorder. Conversely, fat, red meat, alcohol, and coffee may aggravate the condition. As the authors promptly state, all these associations should not be interpreted causally and may be confounded by numerous factors, including social and environmental factors and levels of physical activity. The authors alert that experimental, prospective, randomised studies are required to better understand the relationships between nutritional habits and gynaecological disorders.

## References

[1] Kuller L.H., Pettee Gabriel K.K., Kinzel L.S., Underwood D.A., Conroy M.B., Chang Y., Mackey R.H., Edmundowicz D., Tyrrell K.S., Buhari A.M. (2012). The Women on the Move Through Activity and Nutrition (WOMAN) study: Final 48-month results. Obesity.

[2] Kim Y.J., Tamadon A., Park H.T., Kim H., Ku S.Y. (2016). The role of sex steroid hormones in the pathophysiology and treatment of sarcopenia. Osteoporos. Sarcopenia.

[3] Fuentes N., Silveyra P. (2019). Estrogen receptor signaling mechanisms. Adv. Protein Chem. Struct. Biol..

[4] Nosaka N., Tsujino S., Honda K., Suemitsu H., Kato K., Kondo K. (2021). Effect of ingestion of medium-chain triglycerides on substrate oxidation during aerobic exercise could depend on sex difference in middle-aged sedentary persons. Nutrients.

[5] Amirato G.R., Borges J.O., Marques D.L., Santos J.M.B., Santos C.A.F., Andrade M.S., Furtado G.E., Rossi M., Luis L.N., Zambonatto R.F. (2021). L-Glutamine supplementation enhances strength and power of knee muscles and improves glycemia control and plasma redox balance in exercising elderly women. Nutrients.

[6] Dechichi J.G.C., Mariano I.M., Giolo J.S., Batista J.P., Amaral A.L., Ribeiro P.A.B., De Oliveira E.P., Puga G.M. (2020). Isoflavone supplementation does not potentiate the effect of combined exercise training on resting and ambulatory blood pressure in non-obese postmenopausal women: A randomized double-blind controlled trial-A pilot study. Nutrients.

[7] Waliłko J., Bronowicka P., He J., Brytek-Matera A. (2021). Dieting and disinhibited eating patterns in adult women with normal body weight: Does rumination matter?. Nutrients.

[8] Dos Santos Fechine C.P.N., Monteiro M.G.C.A., Tavares J.F., Souto A.L., Luna R.C.P., Da Silva C.S.O., Da Silva J.A., Dos Santos S.G., De Carvalho Costa M.J., Persuhn D.C. (2021). Choline metabolites, hydroxybutyrate and HDL after dietary fiber supplementation in overweight/obese hypertensive women: A metabolomic study. Nutrients.

[9] De Lorenzo A., Martinoli R., Vaia F., Di Renzo L. (2006). Normal weight obese (NWO) women: An evaluation of a candidate new syndrome. Nutr. Metab. Cardiovasc. Dis..

[10] Kapoor N., Furler J., Paul T.V., Thomas N., Oldenburg B. (2019). Normal weight obesity: An underrecognized problem in individuals of south asian descent. Clin. Ther..

[11] Haghighat N., Ashtary-Larky D., Bagheri R., Wong A., Cheraghloo N., Moradpour G., Nordvall M., Asbaghi O., Moeinvaziri N., Amini M. (2021). Effects of 6 months of soy-enriched high protein compared to eucaloric low protein snack replacement on appetite, dietary intake, and body composition in normal-weight obese women: A randomized controlled trial. Nutrients.

[12] Hubert P.A., Mahoney M., Huedo-Medina T.B., Leahey T.M., Duffy V.B. (2021). Can assessing physical activity liking identify opportunities to promote physical activity engagement and healthy dietary behaviors?. Nutrients.

[13] Bąk-Sosnowska M., Gruszczyńska M., Skrypnik D., Grzegorczyn S., Karolkiewicz J., Ratajczak M., Mądry E., Walkowiak J., Bogdański P. (2021). Type of physical training and selected aspects of psychological functioning of women with obesity: A randomised trial. Nutrients.

[14] Rocha-Rodrigues S., Sousa M., Lourenço Reis P., Leão C., Cardoso-Marinho B., Massada M., Afonso J. (2021). Bidirectional interactions between the menstrual cycle, exercise training, and macronutrient intake in women: A review. Nutrients.

[15] Afrin S., AlAshqar A., El Sabeh M., Miyashita-Ishiwata M., Reschke L., Brennan J.T., Fader A., Borahay M.A. (2021). Diet and nutrition in gynecological disorders: A focus on clinical studies. Nutrients.

